# Microneedle Array Patch Made of Kangfuxin/Chitosan/Fucoidan Complex Enables Full-Thickness Wound Healing

**DOI:** 10.3389/fchem.2022.838920

**Published:** 2022-01-26

**Authors:** Xixi Yu, Caixia Wang, Yuanfei Wang, Longhao Li, Xiang Gao, Tingting Zhu, Pugen An, Zhaojian Meng, Wanchun Wang, Tong Wu, Yuanping Hao

**Affiliations:** ^1^ Qingdao Stomatological Hospital Affiliated to Qingdao University, Qingdao, China; ^2^ Department of Stomatology, School of Stomatology of Weifang Medical University, Weifang, China; ^3^ Department of Cosmetic and Plastic Surgery, Affiliated Hospital of Qingdao University, Qingdao Medical College, Qingdao University, Qingdao, China; ^4^ Shandong Key Laboratory of Textile Materials for Healthcare, Collaborative Innovation Center for Eco-textiles of Shandong Province, Ministry of Education, Qingdao, China

**Keywords:** chitosan, fucoidan, kangfuxin, microneedle array patch, wound healing

## Abstract

Skin wound caused by external injury is usually difficult to be cured by conventional topical administration because of its poor drug diffusion across the stratum corneum. It has been recognized that stratum corneum is the major obstacle for transdermal drug delivery. To address this issue, microneedles (MNs) have been developed to penetrate the stratum corneum of the skin and then form micron-sized pores between the epidermis and the dermis layers. As such, biomacromolecule drugs and/or insoluble drug molecules can be allowed for effective transdermal penetration. A multifunctional microneedle array patch that can avoid wound infection and promote tissue remolding has important value for wound healing. Among others, marine polysaccharides have attracted much attention in multifarious biomedical applications due to their excellent (bio)physical and chemical properties. Herein, we developed a microneedle array patch using a blend of kangfuxin (KFX), chitosan (CS), and fucoidan (FD), named KCFMN, for accelerating full-thickness wound healing. The traditional Chinese medicine KFX extracted from *Periplaneta americana* (PA) has effective bio-functions in promoting wound healing. The macro-/micro-morphology and (bio)physicochemical properties of such composite microneedles were also studied. We showed that the KCFMN patch displayed noticeable antibacterial properties and good cytocompatibility. In particular, the KCFMN patch significantly accelerated the wound healing development in a full-thickness wound in rats by improving the epithelial thickness and collagen deposition. Thus, this versatile KCFMN patch has great prospects as a dressing for full-thickness wound healing.

## Introduction

The skin is the largest sensory organ of the human body, and it is also the main barrier against harmful substances and microorganisms to protect tissues and organs and maintain homeostasis (He et al., 2021). Large-area full-thickness skin wounds affected by skin burns, scalds, trauma, and surgical trauma occur frequently, resulting in infections and scars, which lead to wounds that are difficult to heal and seriously affect individual’s health (Gorantla et al., 2021). Wound healing is a compound multi-level physiological process, which can be separated into four overlapping but distinct stages: hemostasis, inflammation, new tissue formation, and remodeling (Gurtner et al., 2008). In the past few decades, a fact has been proved that wound dressing can be used as a physical barrier to achieve wound healing (Yergoz et al., 2017; Jahromi et al., 2018; Zhu et al., 2018; Hajilou et al., 2020). However, traditional wound dressing materials, such as gauze, hydrogels, sponges, and nanofibers are less efficient in inhibiting bacterial infection and promoting wound healing (Zhao et al., 2017; Chen G. et al., 2018; Chen H. et al., 2018; Yin et al., 2018; Lumbreras-Aguayo et al., 2019; Chen et al., 2020; Dong et al., 2020; Hao et al., 2020; He et al., 2020; Li et al., 2020; Montaser et al., 2020; Qian et al., 2020; Yang et al., 2020; Qian et al., 2021a; Qian et al., 2021b; Dong and Guo, 2021; Hao et al., 2021; He et al., 2021; Wang et al., 2021; Yin et al., 2021; Zheng et al., 2021). In addition, most present patches still have some restrictions: 1) the effect of transdermal delivery across the stratum corneum is poor; 2) the patches have insufficient mechanical strength and are easy to be damaged; 3) the patches have low adhesion and are easy to fall off; and 4) the dressing is thick, with poor adhesion, low air permeability, and foreign body sensation (Zheng et al., 2021). Therefore, it is urgent to develop new approaches for full-thickness wound repair.

Microneedles (MNs) are a new type of physical penetration-enhancing technology, which have many advantages such as the capability to pass through the stratum corneum painlessly, minimal invasiveness, simple production, and convenient administration. Importantly, MNs can bypass the first pass metabolism and can directly enter the systemic circulation (Amani et al., 2021). Thanks to these merits, MNs have proven their value in the medical fields, including tumor treatment, vaccine injection, and sample collection (Kim et al., 2018; Moreira et al., 2019; Samant et al., 2020; Guo et al., 2021; Sheng et al., 2021). According to the demand, various types of MNs have been discovered for the transfer of therapeutic drugs. The types of MNs used to provide treatment include dissolving MNs, solid MNs, hollow MNs, and coated MNs (Gorantla et al., 2021). Among them, dissolving microneedle is a disposable preparation, which is composed of drug and polymer. After being inserted into the skin, the drug is released into the skin layer and the patch is degraded under the action of a variety of enzymes, especially lysozymes (Lee et al., 2008; Ita, 2017; Waghule et al., 2019). Moreover, the choice of polymers is one of the most important steps. The reasons include that the polymer must have biodegradable properties in addition to the strength to penetrate the skin layer, and it must not react with the encapsulated drugs.

Nowadays, Chinese medicine mainly obtained from natural medicines, including botanicals, animals, and insect medicines, which is widely practiced and regarded as one of the alternatives for various wound treatments (Ahn et al., 2019). Chitin is widely distributed in nature. The shells of marine arthropods such as shrimps and crabs are rich in chitin. Chitosan (CS) is a derivative of arthropod chitin, which is a positively charged polyelectrolyte in solution and has a strong adsorption. Chitosan molecules contain amino groups and are alkaline (Chi et al., 2020). MN fabrication using CS has increased meaningful interest due to its biocompatibility, low toxicity, non-antigenicity, biodegradability, hemostasis, and the ability of film-forming, which enables it to achieve local transdermal delivery of MNs (Amani et al., 2021). Due to its positively charged characteristics, CS can penetrate the cell wall of negatively charged bacteria and eventually cause the overflow of intracellular fluid, which in turn leads to the death of bacteria (Chi et al., 2020; Hao et al., 2020). Fucoidan (FD) are mainly derived from brown seaweed, which has a variety of biological functions, such as anticoagulation, antitumor, anti-thrombosis, antivirus, antioxidation, and enhancement of the body’s immune function, so it is widely used in the field of medicine (Hao et al., 2020). In previous studies, FD-CS hydrogels and CS sponges containing FD were reported as wound healing accelerators (Murakami et al., 2010; Hao et al., 2020).

**FIGURE 1:**
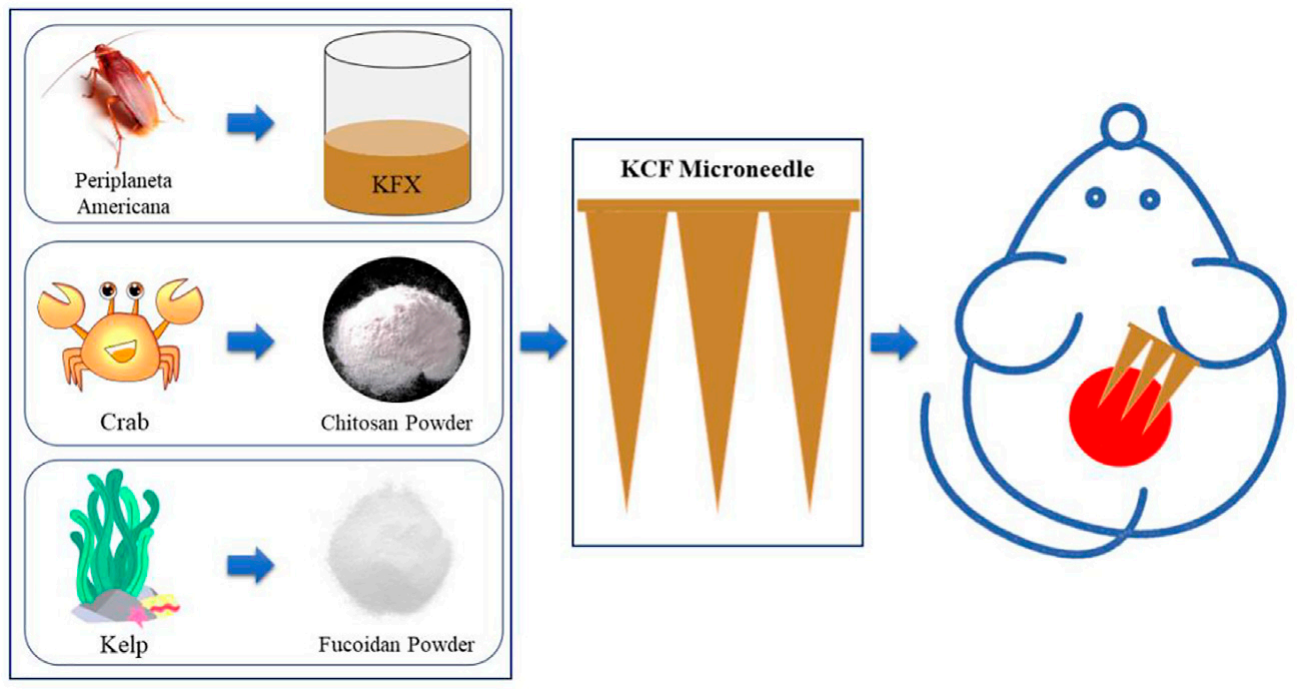
Schematic diagram of sample preparation for KCFMN patches and its application in wound healing.

Particularly, kangfuxin (KFX), a Chinese medicine extracted from *Periplaneta americana* (PA), has been commonly used in the clinical application of various mucosal ulcer treatments (Chen et al., 2016) and has been approved by the China Food and Drug Administration (CFDA) (Z51021834). KFX contains nucleotides, small molecular peptides, and amino acids (Fu et al., 2022). Moreover, it has been confirmed that KFX can promote the growth of new granulation tissue, repair ulcer wounds, enhance immune function, and inhibit bacteria and inflammation (Shen et al., 2017). Recently, Li *et al.* studied the prophylactic effect of KFX and revealed that it can promote wound healing and improve healing through multiple regulations (Li et al., 2019). However, the delivery efficacy of KFX is significantly reduced. Since KFX is a liquid, it cannot completely penetrate the subcutaneous tissue. Herein, we effectively changed the liquid KFX into solid, which is referred as the KCFMN patch (Figure 1).

Based on the earlier summary, it is reasonable to propose a hypothesis that the kangfuxin (KFX)/chitosan (CS)/fucoidan (FD) microneedle (KCFMN) would be administered through the skin to promote epithelial regeneration and inhibit bacterial infection, so as to promote wound healing. The assumption earlier has not been reported. In order to verify the earlier hypothesis, multifunctional MN patches were established through the electrostatic interaction and hydrogen bonding among KFX, CS, and FD in this study. The physical and chemical characteristics of KCFMN patches were characterized by scanning electron microscopy (SEM) and Fourier-transform infrared (FTIR) spectra. In addition, the biocompatibility of the patch was evaluated by cell counting kit-8 (CCK-8) assay. The antibacterial property of KCFMN patches was achieved through antibacterial tests. Last, the full-thickness skin defect rat model and pathological manifestations were used to evaluate the efficiency of wound healing.

## Materials and Methods

### Materials

CS (Mw = 300 kDa and deacetylation degree ≥90%) was obtained from Zhejiang Golden-Shell Pharmaceutical Co., Ltd., China. FD (Mw = 276 kDa, sulfate: 29.65%) was purchased from Qingdao Bright Moon Seaweed Group Co., Ltd. China. KFX was bought from Hunan Kelun Pharmaceutical Co., Ltd. China. Other chemical reagents were taken from Macklin and Sinopharm Chemical Reagent Co., Ltd. China. Deionized water was used in all experiments. Male Sprague–Dawley rats (200 g in weight) were offered by Jinan Pengyue Laboratory Animal Breeding Co., Ltd. China. All rats were treated in strict accordance with the Laboratory Animal Care and Use Guidelines. All the animal care and experimental procedures were evaluated and approved by the Animal Investigation Ethics Committee of Qingdao Stomatological Hospital Affiliated to Qingdao University.

### Fabrication of the KCFMN Patch

The KCFMN patch was fabricated using PDMS molds. CS (4% w/v), FD (0.4% w/v), and acetic acid (1% v/v) were dissolved together with 30 ml of KFX by operating a mechanical stirrer at 700 rpm for two h to prepare the microneedle pre-gel. Under the vacuum state for 30 min, the deionized water was totally filled in the narrowed microcavities of the mold. Excess deionized water was removed. Then, the pre-gel was cast into the mold and completely infilled into the mold by ultrasonic treatment for 30 min. And then the KCFMN patch was dried in a drying oven for 36 h. The resulting KCFMN patch was carefully taken from the mold. Moreover, the procedure of the CFMN patch was carried out in accordance with the steps of KCFMN preparation.

### Scanning Electron Microscopy

To evaluate the physical shape of the KCFMN and CFMN patches, all samples were measured using a SEM (VEGA3, TESCAN, Czech) operated at an acceleration voltage of 10 kV. Samples were sputtered with gold before imaging to increase conductivity.

### Fourier-Transform Infrared Spectroscopy

The Nicolet iN10 FTIR spectrometer (Thermo Fisher Scientific, Waltham, MA, United States) was applied to determine the functional group of the samples (i.e., KFX, CS, FD, CFMN, and KCFMN).

### Biocompatibility Analysis

To examine the biocompatibility of MNs, CCK-8 (Absin Bioscience Inc. China) tests were conducted according to the previous method (Hao et al., 2021; He et al., 2021). Mouse fibroblast cell lines L929 (Cell Culture Center, Shanghai Institute of Life Sciences, Chinese Academy of Sciences) were cultured in Dulbecco’s modified eagle medium (DMEM) containing 10% fetal bovine serum (FBS) with 1% penicillin/streptomycin double antibiotics in an incubator at 37 °C, 5% CO_2_ (DMEM, FBS, 1% penicillin/streptomycin double antibiotics, Biological Industries, Israel). To prepare the extract solutions, equal sized samples of KCFMN and CFMN were sterilized at high temperature in a microwave oven for five min and immersed in one ml of the culture medium and incubated for 24 h. L929 cells were seeded in 24-well plates with a density of 8×10^3^ cells/well for 24 h to ensure attachment. Then the original culture medium was removed, and the cells were washed with PBS solution (Solarbio, China) and divided into three groups. For the control group, a 24-h culture medium was added; for the KCFMN group, 24-h KCFMN extract solution was added; and for the CFMN group, 24-h CFMN extract solution was added. The CCK-8 tests were measured on days 1, 3, and 5, and each group had five parallels. The culture medium was replaced with 100 μL fresh medium. Eventually, cell viability was obtained by CCK-8 assay with a microplate reader (SynergyH1/H1M, Bio-Tek, China).

### Antibacterial Test *In Vitro*

Antibacterial efficacy of the KCFMN and CFMN patches were tested by the colony counting method (Feng et al., 2021). Gram-positive *Staphylococcus aureus* (*S. aureus*) and Gram-negative *Escherichia coli* (*E. coli*) were chosen to evaluate the antimicrobial ability of MNs. It was performed according to the reference (Hao et al., 2020). Two kinds of bacteria were cultured in Luria–Bertani (LB) and tryptic soy broth (TSB) agar plates, separately. Subsequently, the MNs were sterilized at high temperature in a microwave oven for five min, and then all patches were briefly washed with PBS. Afterward, 3 ml bacterial suspension was mixed with equal-sized sample KCFMN and CFMN patches and incubated at 37 °C for 24 h, respectively. Then, after six-fold serial dilution, 10 μL of each bacterial suspension was spread with an agar plate. The plates were then inverted and kept at 37 °C in an incubator for 24 h. Thereafter, colonies were counted on each plate. The experiment comprised three replicates, and the results were expressed as kill%:

$$\mathrm{Kill}\%=\frac{\mathrm{cell}\;\mathrm{count}\;\mathrm{of}\;\mathrm{cuntrol}-\mathrm{survior}\;\mathrm{count}\;\mathrm{with}\;\mathrm{MNs}}{\mathrm{cell}\;\mathrm{count}\;\mathrm{of}\;\mathrm{cuntrol}}\times 100\%.$$

### Wound Healing Study

To further evaluate the *in vivo* effect of KCFMN patches for wound healing, a rat skin circular full-thickness wound model was established (Hao et al., 2020; He et al., 2021). Specifically, healthy male SD rats were first anesthetized by intraperitoneal injection of 1% pentobarbital sodium (Sigma-Aldrich). After their backs were shaved, a 1.0 cm diameter full-thickness skin excision was created on the back of the rat. Then, the rats were randomly divided equally into five groups: the KCFMN, the CFMN, the KCF-Film, the KFX, and the control group. Each group included six rats, and the rats were put back to cages with free food and water after waking from anesthesia. Wounds on individual rats were photographed digitally on days 0, 3, 5, 7, and 9 post-wounding. Furthermore, the wounds were measured with a rule. All rats were killed to analyze on day 9. The wound areas were calculated and quantified using ImageJ software. The closure area of the wound (A_wc_) was calculated as follows:

$$A_{\mathrm{WC}}\left(\%\right)\frac{A0-A}{A0}\times 100\%,$$

where A0 is the initial wound area and A is the wound area at the indicated times.

### Histopathology Assay

Skin tissues were harvested on day 9 and fixed overnight in 4% buffered paraformaldehyde and embedded in paraffin. After the samples were sectioned, hematoxylin–eosin staining (H&E) and Masson’s trichrome staining were performed. H&E samples were observed to assess the epidermal thickness. Collagen synthesis was detected by Masson’s trichrome staining.

### Statistical Analysis

All data were shown as mean ± standard deviation (SD). Statistical analysis was carried out using GraphPad Prism 9 (GraphPad software). Statistical analysis was performed using one-way analysis of variance (ANOVA). The statistically significant *p* values were labeled as follows: *p < 0.05, **p < 0.01, ***p < 0.001, and ****p < 0.0001.

## Results and Discussion

### Preparation and Characterization of KCFMN Patches

**FIGURE 2:**
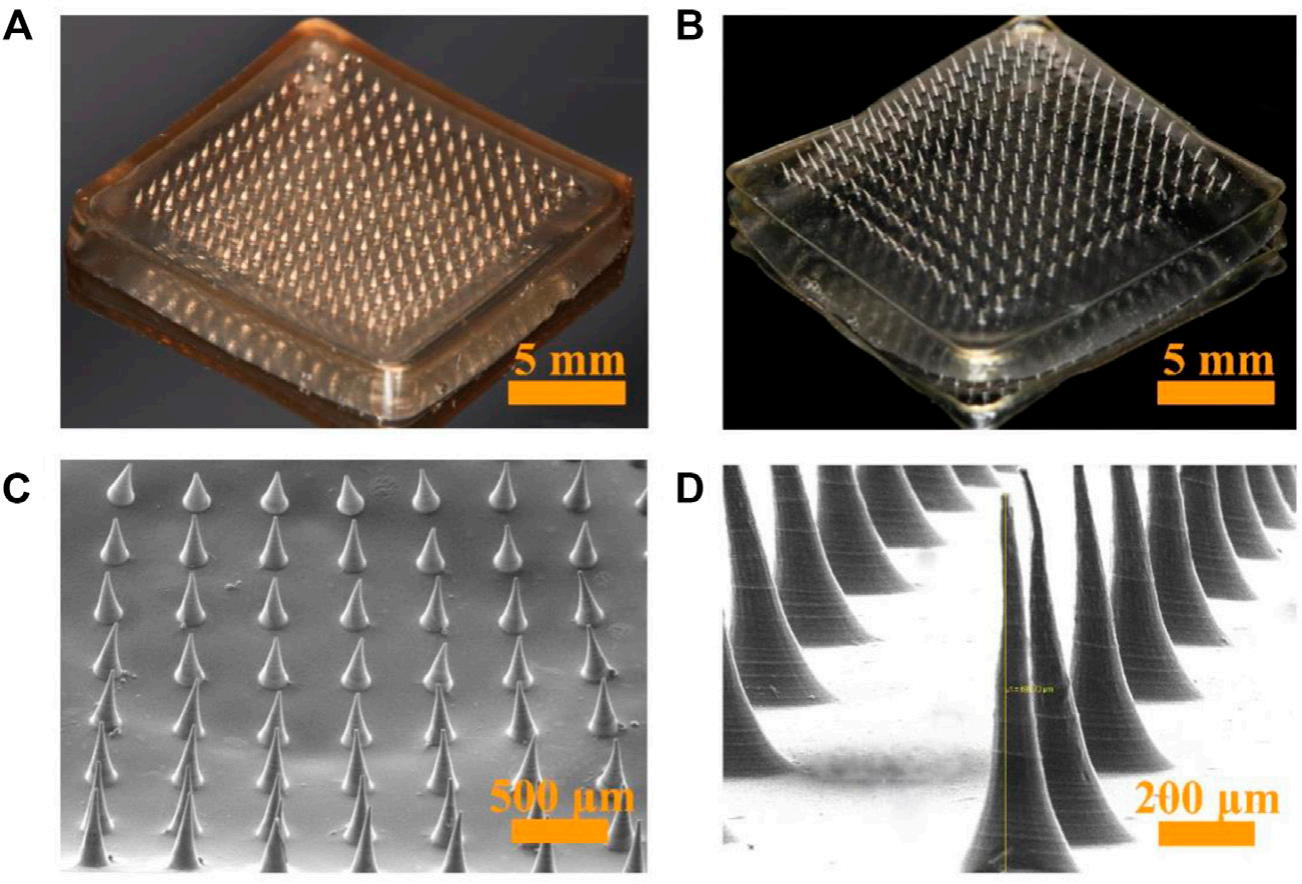
Optical and SEM photographs of KCFMN and CFMN. **(A)** Optical photograph of KCFMN. **(B)** Optical photograph of CFMN. **(C)** SEM photograph of KCFMN. **(D)** Magnified SEM photograph of the individual MN. Scare bars: 5 mm in **(A)** and **(B)**, 500 μm in **(C)**, 200 μm in **(D)**.

KCFMN patches were fabricated by a micro-molding technique. In short, a predetermined amount of KFX, CS, and FD was mixed and stirred to form a pre-gel solution, which were put into the microcavity of the mold, and ultrasonicated for 30 min. Finally, the pre-gel was dried using a drying oven for 36 h. The fabricated KCFMN and CFMN patches were arranged in a 15 × 15 MN arranged on a 15 × 15 mm^2^ support base (Figures 2A,B). KCFMN patches showed light brown because of the KFX (Figure 2A). The MNs exhibited a cone shape with a 5-μm-diameter tip, 700 μm height, and 300 μm base diameter as shown in the SEM images (Figures 2C,D). The microstructure of MNs was further detected by SEM (Figure 2D). We found that the KCFMN patch had no pores on the surface, which is different from previous studies (Chi et al., 2020). The reason may be that CS is not cured with NaOH.

The drug KFX loaded in this study is liquid, in order to prevent the pore drug amount from being too small and can be quantified better; therefore, CS and FD are dissolved directly with KFX to better quantify and ensure that KFX has sufficient drug amount. In order to manufacture KCFMN patches with perfect qualities, the solidification was initially adjusted. Thus, the addition of FD to the solution is beneficial to increase consistency and solidification of the solution, and thereby improve the mechanical strength of KCFMN patches. According to previous research, 4% CS and 0.4% FD are selected to fabricate KCFMN patches in the following experiment (Chi et al., 2020; Hao et al., 2020; Hao et al., 2021; Zheng et al., 2021).

**FIGURE 3:**
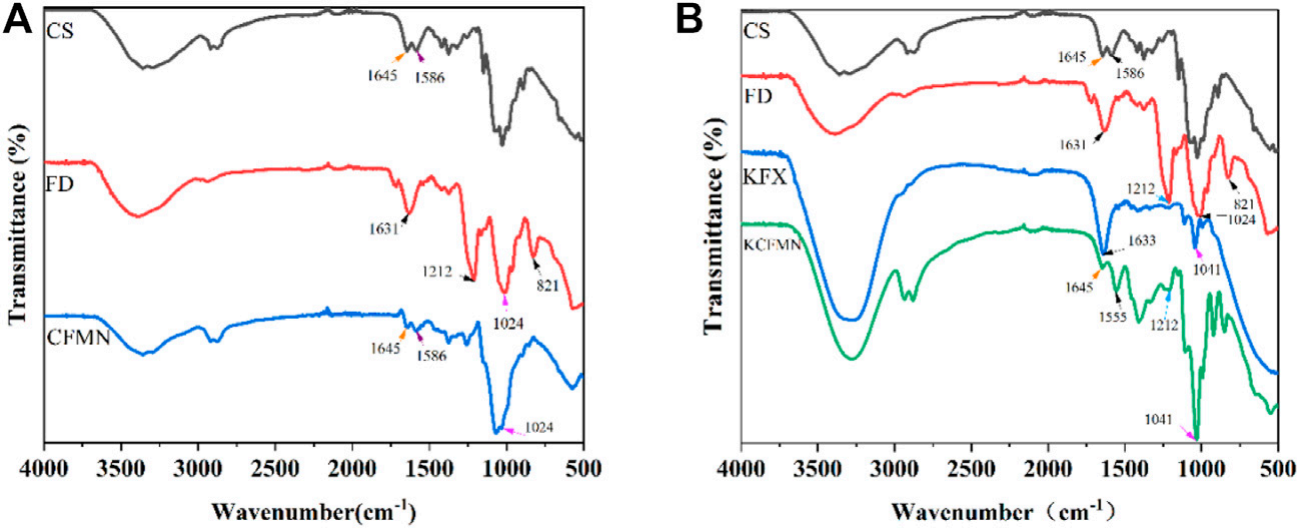
**(A)** FTIR spectra of CS, FD, and CFMN **(B)** FTIR spectra of CS, FD, KFX, and KCFMN.

FTIR spectra of KFX, CS, FD, CFMN, and KCFMN are shown in Figure 3. FTIR spectra of CS showed characteristic peaks at 1,645 and 1,586 cm^−1^ appeared to -C=O stretching and -NH bending vibration, respectively (Don et al., 2006; Hao et al., 2021). The FD showed characteristic peaks at 821 and 1,212 cm^−1^corresponding to the C-O-S stretching (galactose-4-sulfate) and S=O asymmetric stretching (sulfate groups) of sulfate groups, respectively (Manivasagan et al., 2019; Hao et al., 2021). The spectra of CFMN included the characteristic peaks of CS (1,645 and 1,586 cm^−1^) and FD ( 1,024 cm^−1^) and the spectra of KCFMN included the characteristic peaks of CS (1,645 cm ^−1^), FD (1,041 cm^−1^), and KFX (1,212 cm^−1^), respectively, which was revealed that composite KCFMN patches were manufactured without affecting the biologically active functional groups of the individual components in the physical mixing process (Hao et al., 2020; Hao et al., 2021).

### Evaluation of the Biocompatibility and Antibacterial Ability of KCFMN Patches

**FIGURE 4:**
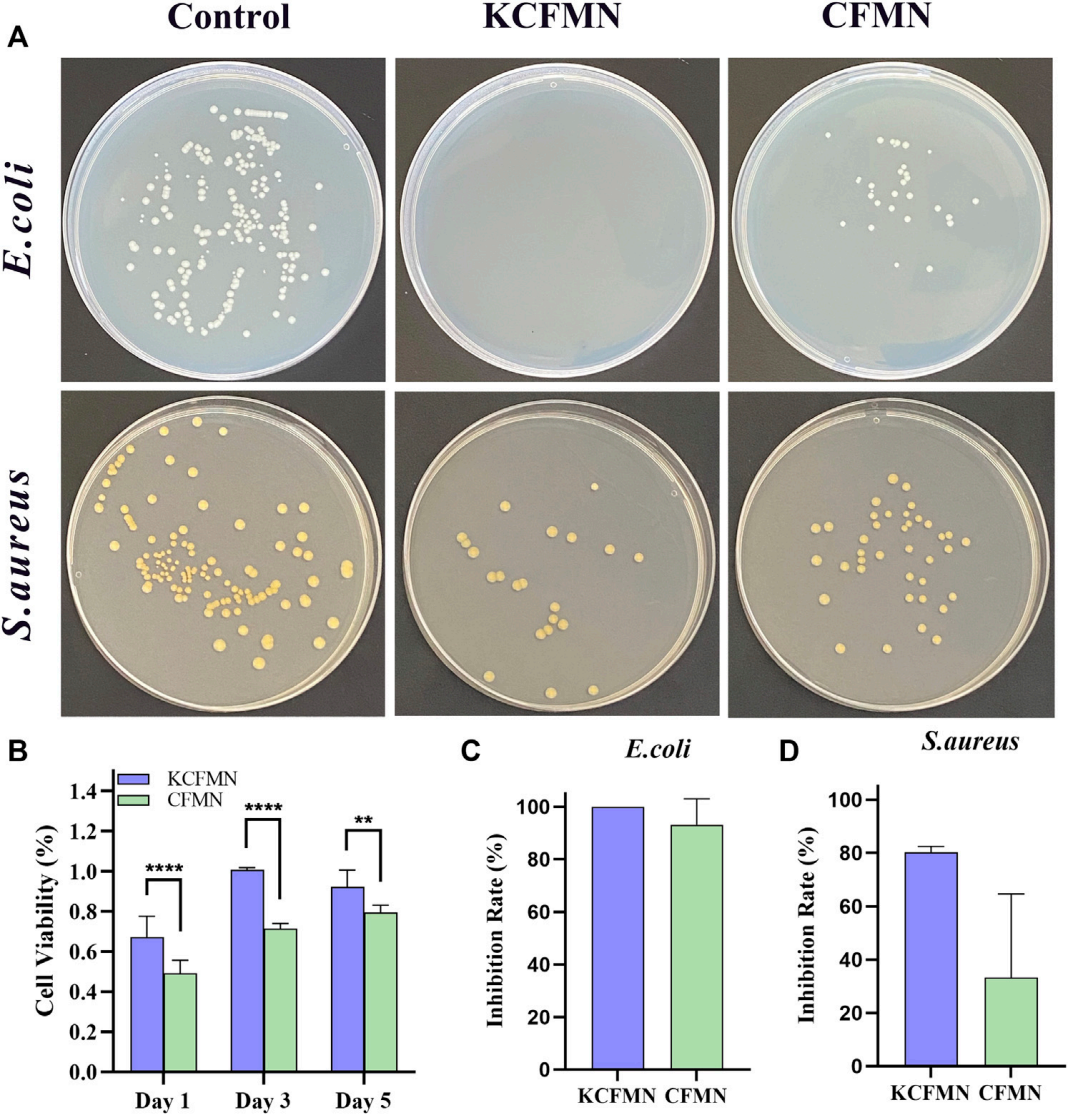
**(A)** Photographs of clones on agar plates after co-cultured with control, KCFMN, and CFMN. **(B)** Cytocompatibility analysis of the KCFMN and CFMN. The cell viability of MNs on day 1, 3, 5 (**p < 0.01 and ****p < 0.0001). **(C)** Inhibition rate of *E. coli* co-cultured with KCFMN and CFMN, respectively. **(D)** Inhibition rate of *S. aureus* co-cultured with KCFMN and CFMN, respectively.

Since KCFMN patches are in direct contact with the wound surface, biocompatibility is very important for the MNs, which can avoid damage caused by its toxic ingredients to the wound (Margolis et al., 2018). To evaluate the biocompatibility of KCFMN patches, L929 cells were co-cultured with extract solutions of MNs. Additionally, CCK-8 assays were carried out to evaluate cell viability (Figure 4B). After days 1, 3, and 5 of culture, the cell viability of KCFMN patches was higher than that of the CFMN patches. This result showed that KCFMN patches had good biocompatibility and could meet the basic requirements for subsequent animal experiments used as wound dressing materials.

The ideal wound dressing not only acts as a barrier to prevent foreign bacterial infections but also has antibacterial properties. The colony forming unit (CFU) test was used to test the antibacterial ability of KCFMN patches against *S. aureus* and *E. coil* (Hao et al., 2020; He et al., 2021). As shown in Figure 4A, these pictures qualitatively showed that the number of colonies on the KCFMN and CFMN groups were significantly reduced compared with the control group. The inhibition rate was calculated from the number of colonies. It can be seen from Figures 4C,D that the inhibition rate of KCFMN on *E. coli* is close to 100%, and the inhibition rate on *S. aureus* reaches 80%, indicating that the KCFMN patch exhibited antibacterial activity against *E. coil* is more durable than that of *S. aureus*. That is probably due to the sterilization effect of KFX on *E. coil* is better than that of *S. aureus*, but the specific mechanism of action is unclear (Shen et al., 2017; Hao et al., 2020). In summary, the excellent antibacterial activity and biocompatibility of the KCFMN patch showed the potential to effectively prevent wound infection with bacteria.

### Evaluation of Full-Thickness Skin Excisional Wound Healing *In Vivo*

**FIGURE 5:**
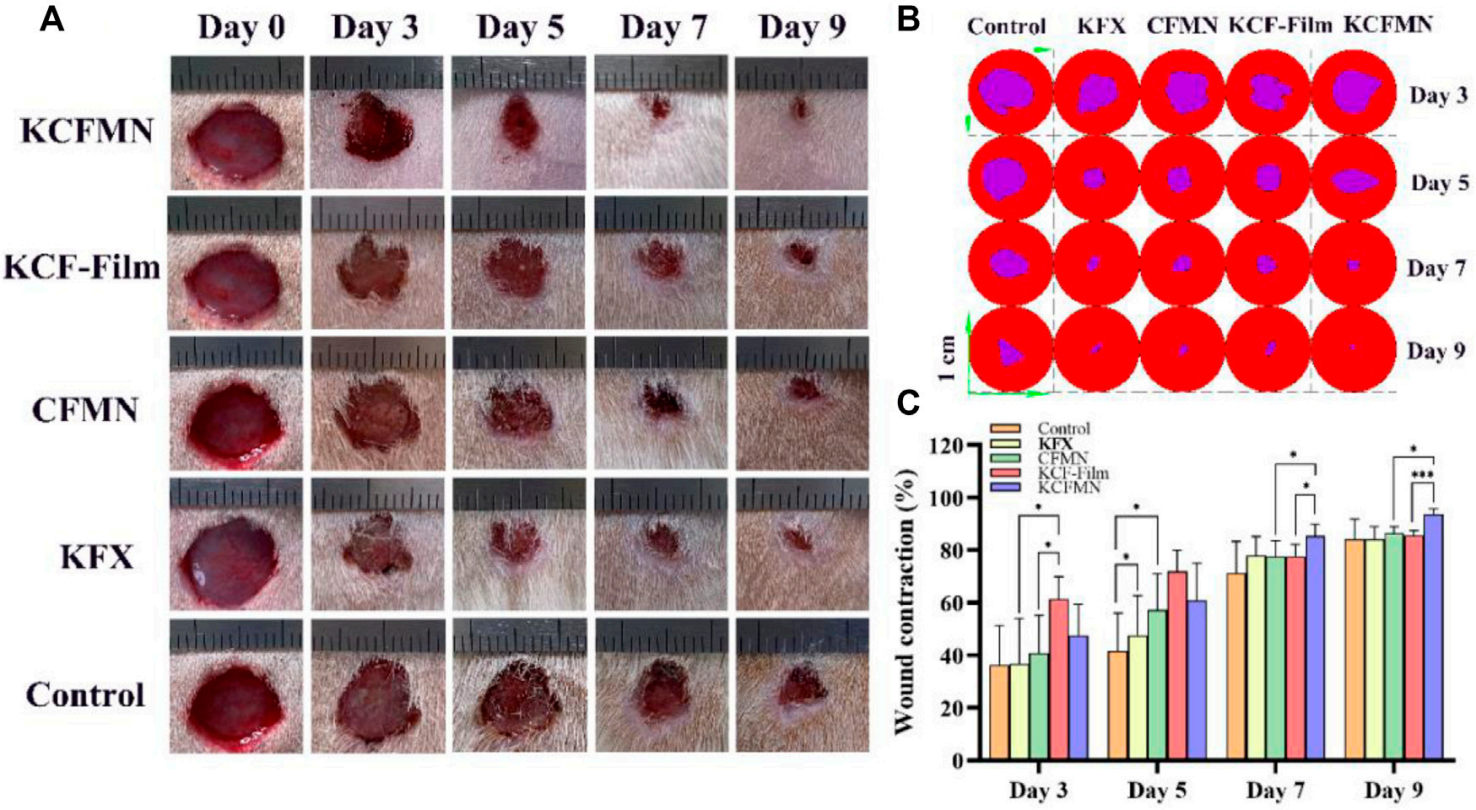
**(A)** Representative images of the wounds treated by KCFMN, KCF-Film, CFMN, KFX, control group on days 0, 3, 5, 7, and 9. **(B)** Traces of wound closure for 3–9 days. **(C)** Wound contraction for 3–9 days (*p < 0.05 and ***p < 0.001).

In ancient China, people usually utilized Chinese medicine mainly obtained from natural medicines, including botanicals, animals, and insect to promote wound healing (He et al., 2021). Particularly, KFX is a Chinese medicine extracted from PA, which has been widely used for various mucosal ulcer treatments. To demonstrate the excellent wound healing ability of KCFMN patches, a full-thickness skin defect model made on rats was tested. Subsequently, KCFMN, KCF-Film, CFMN, and KFX were used in the wound areas, respectively. Rats performed with PBS solution were used as the control group. Data from the five different groups were recorded on days 0, 3, 5, 7, and 9 for further detailed analysis. Qualitative analysis of the photos (Figure 5A) and their traces (Figure 5B) showed that the wound healing effect of KCFMN patches was higher than that of the KCF-Film, CFMN, KFX, and control groups.

Quantitative analysis of the wound contraction (Figure 5C) suggests that the wound healing effect treated with the KCF-Film group was best on days 3 and 5, which was significantly higher than the CFMN, KFX, and control groups. Moreover, on day 5, the KFX group had statistical significance with the control group. This may be the reason that the KCF-Film and KFX can directly and closely contact the wound surface, causing the drug to act directly, thereby promoting wound healing. However, the KCF-Film has a longer action time than KFX; therefore, the effect of the KCF-Film was better than that of KFX. On days 7 and 9, the KCFMN group was obviously different compared with the KCF-Film and the CFMN group, which indicated that the ability of the KCFMN group to accelerate wound healing was higher than that of the KCF-Film and the CFMN group. The reason may be that in the first five days, the KCF-Film can directly act on the wound at the beginning, so the effect was better than other groups. The healing of the KCFMN group on days 7 and 9 was significantly better than that of the KCF-Film, which may be the slow-release effect of KCFMN under the skin. In addition, the KCFMN group was better than the CFMN group, which was attributed to the healing effect of the KFX.

### Evaluation of H&E and Masson’s Trichrome Staining

**FIGURE 6:**
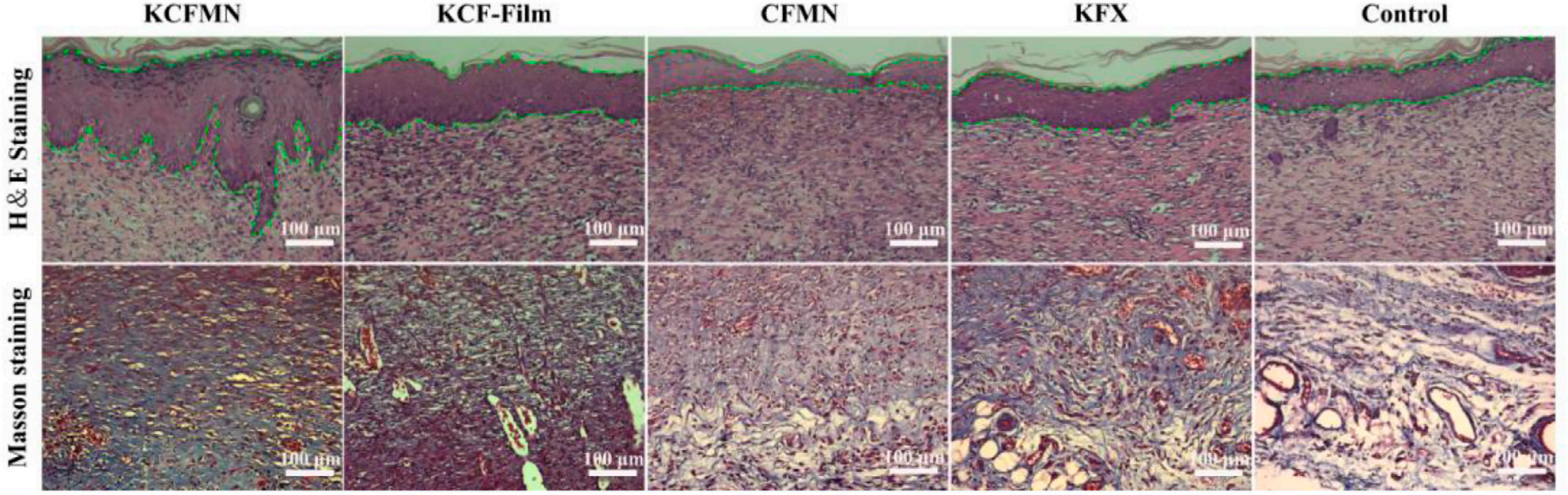
H&E and Masson’s trichrome staining of wound skin tissues. Scale bars represent 100 μm.

H&E and Masson’s trichrome staining were used to analyze the histological status of the wound at the end of healing on day 9 (Figure 6). We were pleasantly surprised to find that the re-epithelialization of the KCFMN group was faster than the other groups. The H&E staining image showed that the thickness of the newly formed epidermis in the KCFMN group was much thicker than that in the other groups. Therefore, it showed that KCFMN had a strong ability to promote wound healing. In addition, the results of Masson’s trichrome staining showed that the KCFMN group had more collagen fibers, and the fibers and collagen were arranged more regularly, which indicated a stronger dermis. This phenomenon might be explained as the KCFMN group can accelerate wound healing by promoting epithelial thickening and increasing collagen deposition.

## Conclusion

In summary, the kangfuxin/chitosan/fucoidan microneedle array patch for promoting wound healing was successfully developed *via* the van der Waals force. KCFMN patches can penetrate the stratum corneum of the skin, promote the penetration of KFX and marine polysaccharide, and overcome the problem of low transdermal penetration of traditional drugs. In addition, KCFMN patches showed better biocompatibility and antibacterial abilities and significantly promoted the closure of full-thickness wounds in rats by remodeling the epithelium. Thus, KCFMN patches have great clinical wound healing potential for these advantages mentioned before.

## Data Availability

The original contributions presented in the study are included in the article/Supplementary Material; further inquiries can be directed to the corresponding authors.
